# Systemic Lupus Erythematosus Presenting With Hemoptysis at Onset in an Elderly Male

**DOI:** 10.7759/cureus.93360

**Published:** 2025-09-27

**Authors:** Tamanna Mohta, Chukwuemeka Umeh, Anushka Chokshi

**Affiliations:** 1 Internal Medicine, Hemet Global Medical Center, Hemet, USA; 2 Internal Medicine, St. Mary Medical Center, Langhorne, USA

**Keywords:** antinuclear antibody, elderly, male, pyrexia, systemic lupus erythematosus

## Abstract

Systemic lupus erythematosus (SLE) is a connective tissue disorder with an unknown cause that primarily impacts women of reproductive age. We present a case of a new-onset elderly male SLE with positive anti-nuclear antibodies (ANAs) and clinical presentations including hemoptysis, fatigue, weight loss, multiple skin and mucosal lesions, and intractable pyrexia. The European League Against Rheumatism guidelines and comprehensive evaluations were used to establish a diagnosis of SLE. The prevalence of SLE in males is lower compared to women, often presenting with negative ANA, which makes diagnosis challenging. An unusual and striking feature in this case was hemoptysis as the initial presenting symptom, a rare manifestation that highlights the need for heightened suspicion. Thus, having a high degree of suspicion as a primary care doctor helps in early diagnosis, improves treatment outcomes, and leads to a better quality of life.

## Introduction

Systemic lupus erythematosus (SLE) is a connective tissue disorder with a striking female predominance, affecting women approximately nine times more often than men, particularly during the reproductive years [1]. It is serologically characterized by autoantibodies targeting self-proteins. The onset and severity of symptoms seem to be influenced by environmental factors and strong underlying genetic factors [1]. Autoimmune diseases prevalent in men typically manifest clinically before the age of 50 and are characterized by acute inflammation. In contrast, autoimmune diseases predominantly affecting women tend to appear clinically later in life and are associated with chronic conditions, fibrosis, and an increased number of autoantibodies [2].

For SLE, it is also believed that hormonal factors are associated with disease pathogenesis; however, an extensive explanation for the rarity of the disease in men remains incompletely understood [3]. Sex hormones have been demonstrated to interact with the immune system, affecting B-cells, T-cells, dendritic cells, and cytokine networks. It is believed that female sex hormones increase autoimmune reactivity and contribute to the immune dysregulation that leads to SLE [4]. Interestingly, SLE in men has been observed to be more common among those with Klinefelter syndrome (i.e., genotype XXY), which further supports a hormonal hypothesis [5].

We report a case of an elderly man with no known diagnosis of SLE who presented with hemoptysis and acute shortness of breath. He was then diagnosed with SLE and showed marked improvement with treatment at the end of his hospital stay.

## Case presentation

A 67-year-old Caucasian male with a medical history of hypertension and pancreatitis presented with a complaint of hemoptysis associated with acute shortness of breath. The patient mentioned that he had dysuria three days before the presentation, which he was self-treating with 1000 mg of aspirin daily for three days. He also endorsed a weight loss of 50 pounds in the past three months associated with poor appetite and abdominal pain, possibly from his pancreatitis, for which he was frequently seen in multiple tertiary care centers. He denied a smoking history or recreational drug use.

On physical examination, he was frail and cachectic, alert, and oriented. Auscultation of the lungs suggested mild crackles in the bilateral lower lobes; no wheezing was appreciated. The abdominal exam was positive for mild epigastric tenderness on deep palpation; however, no distension, guarding, or rebound tenderness was present. He was hemodynamically unstable with hypotension and tachycardia. His oxygen saturation on room air was 60%, which improved to 88% on 40 L Vapotherm. His blood work revealed a hemoglobin level of 5.8 g/dL, along with a white blood cell count of 2.8 x 109/L and a lactic acid level of 12.2 mmol/L (Table 1). Urinalysis did not suggest an ongoing urinary tract infection. The anemia workup revealed microcytic anemia with normal lactate dehydrogenase and haptoglobin levels, suggesting that hemolysis is unlikely. He received two units of packed red blood cells. He was admitted to the intensive care unit for treatment of shock, likely hemorrhagic shock secondary to recent aspirin use and possibly septic shock with an unknown source.

**Table 1:** Pertinent admission laboratory values with autoimmune workup Reference ranges may vary slightly depending on the laboratory. dL: deciliter, µL: microliter, mL: milliliter, IgG: immunoglobulin G, IU: international units, GPL: IgG phospholipid units, DNA: deoxyribonucleic acid

Laboratory test	Patient value	Reference range
White blood cell count	2.6 × 10⁹/L	4.0-11.0 × 10⁹/L
Hemoglobin	5.8 g/dL	13.5-17.5 g/dL (male)
Hematocrit	19.2%	41-53% (male)
Platelet count	104 × 10³/µL	150-400 × 10³/µL
Lactic acid	12.2 mmol/L	0.5-2.2 mmol/L
D-dimer	6573 ng/mL	<500 ng/mL
Anti-nuclear antibody	>1:1280, speckled	<1:40, negative
Anticardiolipin antibody IgG	23 GPL units	<20 GPL units
Double-stranded DNA antibody IgG	11 IU/mL	<10 IU/mL

**Figure 1:**
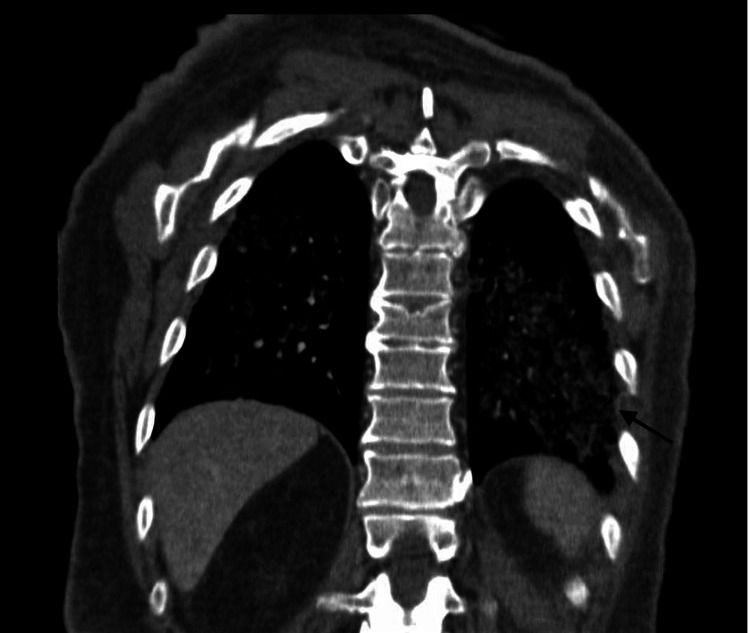
CT angiogram of the chest CT angiogram of chest suggestive of pulmonary edema (arrow) and possible alveolar hemorrhage as a differential diagnosis. CT: computed tomography

Given the history and presentation of acute shortness of breath with hemoptysis, hypotension, and tachycardia, suspicion of pulmonary embolism and malignancy was high, for which a CT angiogram of the chest was ordered. The CT angiogram was suggestive of pulmonary edema and bilateral lower lobe consolidations with a differential of diffuse alveolar hemorrhage (Figure 1).

The CT scan showed no large segment pulmonary embolism. Still, an incidental liver mass in segments 2 and 3, measuring 4.2 x 4.2 x 3.6 cm, was identified, raising concerns for a potential neoplasm or infection. A CT abdomen was ordered, which suggested moderate biliary dilatation in the left hepatic lobe associated with an area of mild hypodensity, along with moderate splenomegaly. Given the CT findings, infectious disease and hematology-oncology specialists were consulted.

The patient’s hemoptysis resolved on day 2 of admission, along with stabilization of their hemoglobin level. He was started on cefepime and vancomycin, and diagnostic tests for fungal and parasitic infections were ordered. A diagnostic panel for autoimmune diseases was also ordered. The hematology-oncology specialist recommended an ultrasound-guided biopsy of the liver mass for further evaluation and management.

Clinically, the patient's condition worsened on day 3, with recurrent spikes of fever reaching up to 102-103°F, which responded briefly to intravenous Tylenol and cooling measures before recurring. He developed purpura on both lower extremities, extending up to the umbilicus, and also developed oral mucosa ulceration. Interventional radiology attempted an ultrasound-guided biopsy of the mass but failed to visualize it on ultrasound. The patient's worsening respiratory condition rendered him unstable for an MRCP, which would have provided better visualization of the hepatic mass.

**Figure 2:**
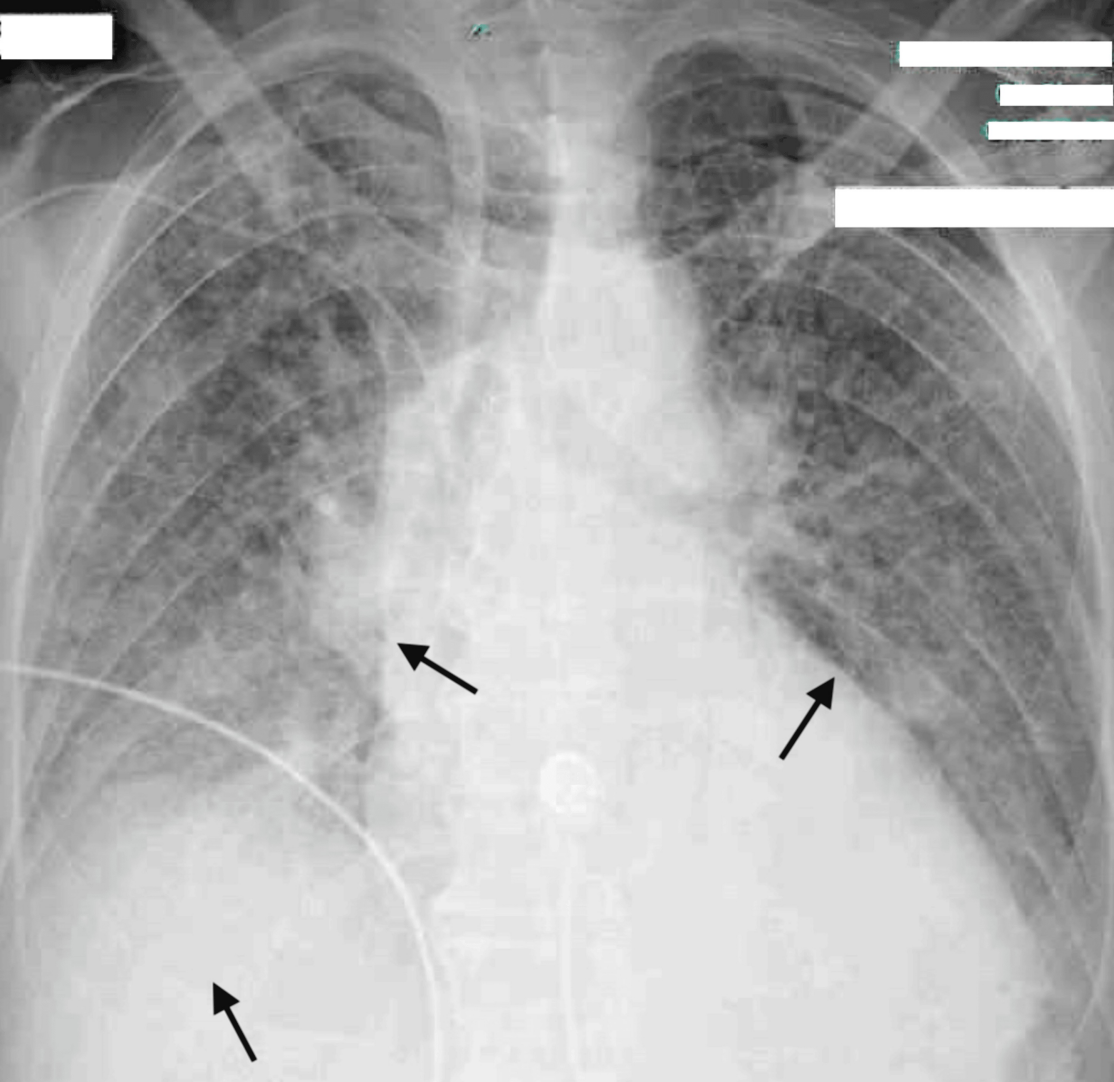
Pre-treatment CXR AP view CXR AP taken before administration of pulse dose steroids. Arrows pointing to the haziness indicate edema. CXR: chest X-ray, AP: anteroposterior

**Figure 3:**
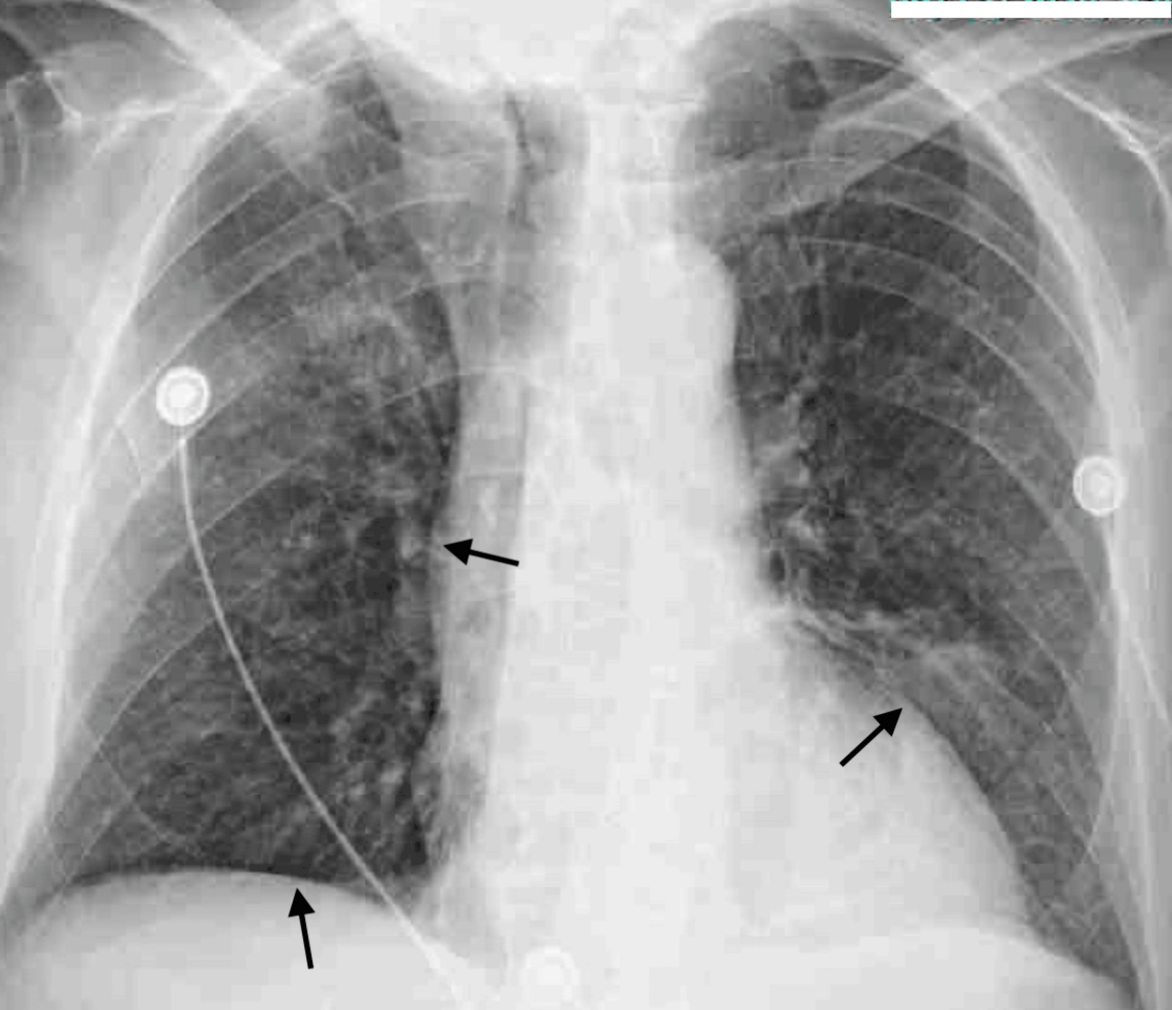
Post-treatment CXR AP view CXR AP done after finishing pulse dose steroids for three days. Arrows indicate improvement in haziness seen previously. CXR: chest X-ray, AP: anteroposterior

On day 4, the anticardiolipin IgG test yielded a positive result, shifting the focus to a possible autoimmune source of his symptoms. Throughout the hospital course, the anti-nuclear antibody (ANA) test was positive in a speckled pattern with a titer of >1:1280 (Table 1). A multidisciplinary team, including specialists from critical care, hematology-oncology, and infectious diseases, decided to give the patient a trial of pulse-dose steroids. Following the administration of 1 g methylprednisolone for three days, the patient showed significant improvement. His oral lesions and purpura resolved completely, and there was no recurrence of fever. Daily chest radiographs also showed improvement in pulmonary haziness (Figures 2-3).

After 10 days of admission, the anti-double-stranded DNA test, which was sent to an external lab, also yielded a positive result. Based on the 2019 European League Against Rheumatism/American College of Rheumatology (EULAR/ACR) classification criteria for SLE, he scored 27 points (Table 2). While these criteria are intended for classification rather than diagnosis, in the appropriate clinical context, they supported the diagnosis of new-onset SLE with flare.

**Table 2:** Points scored based on the EULAR/ACR criteria Points scored are based on the 2019 EULAR/ACR classification criteria for SLE [6]. EULAR: European League Against Rheumatism, ACR: American College of Rheumatology, SLE: systemic lupus erythematosus, DNA: deoxyribonucleic acid

Clinical/immunologic domain	Points	Points for our patient
Fever	2	2
Non-scarring alopecia	2	0
Oral ulcers	2	2
Subacute cutaneous or discoid lupus	4	0
Acute cutaneous lupus	6	0
Synovitis or tenderness in at least 2 joints	6	0
Delirium	2	0
Psychosis	3	0
Seizure	5	0
Pleural or pericardial effusion	5	5
Acute pericarditis	6	0
Leukopenia	3	3
Thrombocytopenia	4	4
Autoimmune hemolysis	4	0
Proteinuria >0.5 g/24 hr	4	0
Class II or V lupus nephritis	8	0
Class III or IV lupus nephritis	10	0
Antiphospholipid antibody domain	2	2
Low C3 or low C4	3	3
Low C3 and low C4	4	0
Anti-ds DNA antibody	6	6
Anti-Sm antibody	6	0

Our hospital did not have an on-call rheumatologist; thus, with the help of the critical care physician, a tapering dose of steroids was started, and later hydroxychloroquine was added. He showed significant improvement over the next five days, began tolerating oral nutrition, and regained physical strength. He was discharged after 16 days of hospital stay with outpatient rheumatology follow-up for SLE and antiphospholipid syndrome.

## Discussion

SLE is a clinically heterogeneous autoimmune disease of unknown etiology, characterized serologically by autoantibodies targeting self-proteins. The incidence and prevalence of SLE vary among different ethnicities, minority groups, and genders. In a systematic review of the worldwide incidence and prevalence of SLE, North America had the highest prevalence, and northern Australia had the lowest prevalence [7]. People of African ethnicity had the highest estimates. Women were more frequently affected than men across all age and ethnic groups [8].

Females of several mammalian species exhibit higher antibody responses than males [9]. Hughes et al. have reported genetic differences in SLE with significant gene-sex interactions in the human leukocyte antigen gene region and IRF5 gene region [10]. Their study also reported a genetic effect distinct to women in the KIAA1542 gene. Their findings suggested that sex differences in SLE could also be attributed to factors other than the X chromosome and hormonal differences [10].

SLE presents with similar symptoms in men and women, particularly joint pain, fatigue, and skin rash. However, diagnosis in men is significantly delayed due to lower clinical suspicion. Some studies show that men can have more complex courses and that renal impairment and vascular disease are more common in them [2]. On the other hand, Murphy and Isenberg reported evidence of a reduced incidence of alopecia, malar rash, and arthralgia/arthritis in men at presentation and throughout the disease course [11].

Our patient was diagnosed based on the 2019 EULAR/ACR guidelines [6], which classify a patient as having SLE if the score exceeds 10. Our patient scored 27 points (Table 2). He was treated with a pulse dose of steroid for an acute SLE flare and then discharged on hydroxychloroquine. Glucocorticoids and antimalarial drugs are the cornerstone of lupus management, supplemented by immunosuppressive or biologic drugs. Current studies also recommend using hydroxychloroquine, when not contraindicated, in all SLE patients, as it not only controls disease activity but also reduces damage accumulation and mortality rates [12].

This case report aims to raise awareness among primary care physicians to suspect SLE in patients who present with systemic symptoms irrespective of age and gender. It underscores the importance of a multidisciplinary approach in overcoming uncertainties and ensuring optimal management, which is vital for reducing morbidity and mortality while enhancing patient quality of life.

## Conclusions

This case underscores the importance of maintaining a high index of suspicion for SLE in atypical patient populations, including elderly males presenting with non-specific systemic symptoms such as hemoptysis, fever, mucocutaneous lesions, and weight loss. Given the rarity and often delayed diagnosis of SLE in men, especially those of advanced age, early recognition and prompt initiation of immunosuppressive therapy can lead to favorable outcomes. Our patient demonstrated a remarkable response to steroid therapy and hydroxychloroquine, highlighting the critical role of timely intervention. This report reinforces the value of a multidisciplinary approach and adherence to established diagnostic criteria, such as the EULAR/ACR guidelines, in managing complex and uncommon presentations of SLE. Clinicians, particularly in primary care and emergency settings, should consider SLE in their differential diagnosis regardless of gender or age to improve prognosis and quality of life.
